# Comparison of T1 Mapping on Gadoxetic Acid-Enhanced Magnetic Resonance Imaging With Conventional Functional Liver Reserve Indices and Technetium-99m Galactosyl Serum Albumin Scintigraphy

**DOI:** 10.7759/cureus.70952

**Published:** 2024-10-06

**Authors:** Kei Takase, Kazuhiro Saito, Yu Tajima, Yoichi Araki, Kenji Uchida, Daisuke Hakamata, Katsutoshi Sugimoto, Daisuke Yuunaiyama, Yuki Takara

**Affiliations:** 1 Radiology, Tokyo Medical University, Tokyo, JPN; 2 Gastroenterology and Hepatology, Tokyo Medical University, Tokyo, JPN

**Keywords:** 99mtc-gsa scintigraphy, functional liver reserve, gadoxetic acid, mri, t1 map

## Abstract

Background

Gadoxetic acid (EOB)-enhanced magnetic resonance imaging (MRI) (EOB-MRI) can be used as a one-stop examination for detecting liver tumors and evaluating liver function.

Purpose

The study aimed to assess the functional liver reserve (FLR) using the T1 map from the hepatobiliary phase of EOB-MRI by conducting a comparison with the results of conventional FLR tests and the technetium-99m (99mTc)-galactosyl serum albumin (GSA) scintigraphy.

Materials and methods

The retrospective data from 43 patients were included in the study. The regions of interest covered the entire liver. The data acquired from each EOB-MRI slice were summed to derive voxel-by-voxel values. The average sum of the T1 values (pre- and post-enhancement), ∆T1, and ∆T1 ratios were calculated. The HH15, LHL15, and LU15 values were calculated from the GSA scintigraphy. The results of conventional FLR tests, such as the indocyanine green retention rate at 15 min (ICGR15), the Child-Pugh classification (CPC), and the albumin-bilirubin (ALBI) and albumin-indocyanine green evaluation (ALICE) scores, were obtained.

Results

The T1 pre- and post-sum values showed a weak correlation with the LHL15 (r=0.36 and 0.38, respectively). A strong correlation was observed between the liver volume and the T1 pre- and post-sum values (r=0.86 and 0.76, respectively). A moderate correlation was observed between the T1 mean and the ALBI and ALICE values (r=0.58 and 0.49, respectively) and between the ∆T1 ratio and the CPC, ALBI, and ALICE values (r=−0.40, 0.58, and −0.55, respectively). The T1 post-sum values showed a moderate correlation with the ALBI scores (r=0.47) and a weak correlation with the ALICE scores (r=0.38). Furthermore, the LU15 values showed a weak correlation with the ICGR15 and model for end-stage liver disease (MELD) scores (r=−0.32 and −0.34, respectively).

Conclusions

Representative indices, such as the T1 mean and ∆T1 ratio, demonstrated a better relationship with conventional FLR indices compared with volumetric radiological indices. Therefore, we propose that the T1 post-sum can be used as an FLR index.

## Introduction

Evaluation of the functional liver reserve (FLR) is important to eliminate the risk of postoperative liver failure [1]. Several indices have been proposed, such as the indocyanine green retention rate at 15 min (ICGR15), Child-Pugh classification (CPC) [1], model for end-stage liver disease (MELD) [2], albumin-bilirubin (ALBI) [3], albumin-indocyanine green evaluation (ALICE) [4], technetium-99m (99mTc)-galactosyl serum albumin (GSA) (99mTc-GSA) scintigraphy [5], and gadoxetic acid (EOB)-enhanced magnetic resonance imaging (MRI) (EOB-MRI) [6,7]. The ICGR15, CPC, MELD, ALBI, and ALICE are conventional indices based mainly on biochemical tests, while the 99mTc-GSA scintigraphy and EOB-MRI are based on radiological imaging. Evaluation of the FLR based on tomographic images is useful because it can be simulated preoperatively. A diseased liver has an inhomogeneous function [8]; therefore, examinations that evaluate the FLR, such as GSA scintigraphy, have the advantage of estimating postoperative remnant liver function. Several authors have previously reported on postoperative remnant liver function [9,10].

Furthermore, EOB-MRI exhibits excellent detection of primary liver and metastatic cancers, and its preoperative usefulness is widely recognized. The EOB is taken to the hepatocytes by transporters and excreted into the bile; therefore, liver function can be evaluated by quantifying the contrast media accumulated in the hepatocytes. Consequently, EOB-MRI can be used as a one-stop examination for liver tumor detection and liver function evaluation [8,11]. Several approaches have been attempted to evaluate liver function [11-16]. Among them, T1 mapping is not dependent on the MRI parameters; therefore, it is highly reliable and reproducible [16]. The T1 value measurement for the entire liver was obtained by the sum of each pixel of the MRI scan to evaluate the FLR. To examine the possibility of FLR assessment by quantifying the EOB taken up by the liver using T1 mapping, the T1 value index obtained from T1 mapping was compared with conventional FLR indices.

## Materials and methods

Participants

This study was conducted according to the Declaration of Helsinki (as revised in 2013). In addition, the Institutional Ethical Review Board of Tokyo Medical University approved this study (approval number: T2020-0330) and waived the requirement for individual consent for the retrospective analysis.

**Figure 1:**
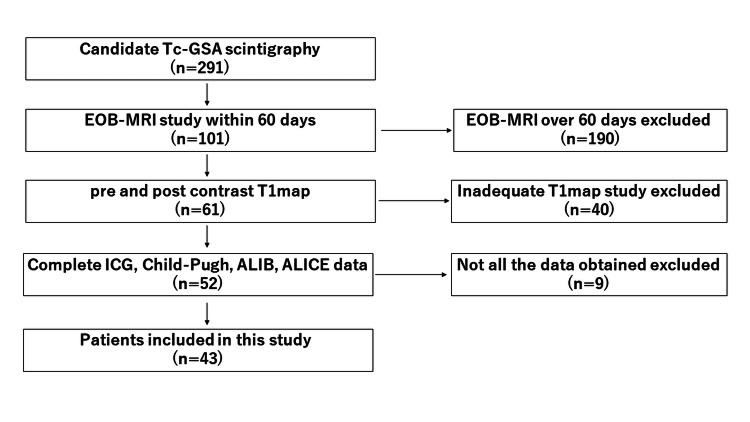
Flowchart showing participant selection details.

Patients were selected from radiological reporting system entries between January 2014 and April 2020 (Figure 1). The inclusion criterion was that GSA scintigraphy and EOB-MRI were performed on the patient. The exclusion criteria were as follows: (1) the time between the GSA scintigraphy and EOB-MRI was ≥60 d; (2) a T1 map was not obtained either before or after administration of the EOB; (3) preoperative portal vein embolization was performed; and (4) the patient's data did not include all the ICGR15, CPC, ALBI, and ALICE values.

GSA acquisition

The GSA single-photon emission computed tomography (SPECT) with high-resolution collimator and CT fusion images were acquired using a Symbia Intevo system (Siemens Healthcare AG, Munich, Germany), which combines SPECT with CT. The GSA scintigraphy was performed on patients after an overnight fast. First, 185 MBq of GSA (Nihon Medi-Physics Co., Ltd., Tokyo, Japan) was injected into the patient's antecubital vein. A bolus injection of radiotracer was followed by 20 mL of saline. Sequential anterior abdominal 64×64 matrix size images, including the liver and heart, were acquired every 30 s for 16 min using a gamma camera's large field of view (FOV). The hepatic SPECT data (60 steps, 15 s/step, 360°, 128×128 matrices) were acquired during the first 20-35 min. The SPECT images were reconstructed with attenuation and scatter correction using the ordered-subset expectation-maximization (OS-EM) algorithm (three iterations, eight subsets) with a 7-8-mm voxel size. Subsequently, CT was performed.

EOB-MRI acquisition

The MRIs were performed using a 3 Tesla (3T) MRI system (MAGNETOM Vida or MAGNETOM Skyra, Siemens AG, Erlangen, Germany); T1-weighted, T2-weighted, diffusion-weighted, and dynamic images were obtained. First, 0.025 mmol/kg of EOB was injected via the patient's antecubital vein. Then, contrast media was injected at 2 mL/s, followed by 40 mL of saline at the same speed. A T1 map was obtained before and 20 min after the injection of the contrast media. The parameters of the T1 map were as follows: TR/TE/FA 5.08 msec/2.34 msec/3°, 15°, matrix 156×256, FOV 380 mm, reduced FOV (RFOV) 81.3%, CAIPIRINHA 4 (2×2), slice thickness 4 mm, gap 0.8 mm, slice per slab 64, and scan time 21 s.

Analysis

**Figure 2:**
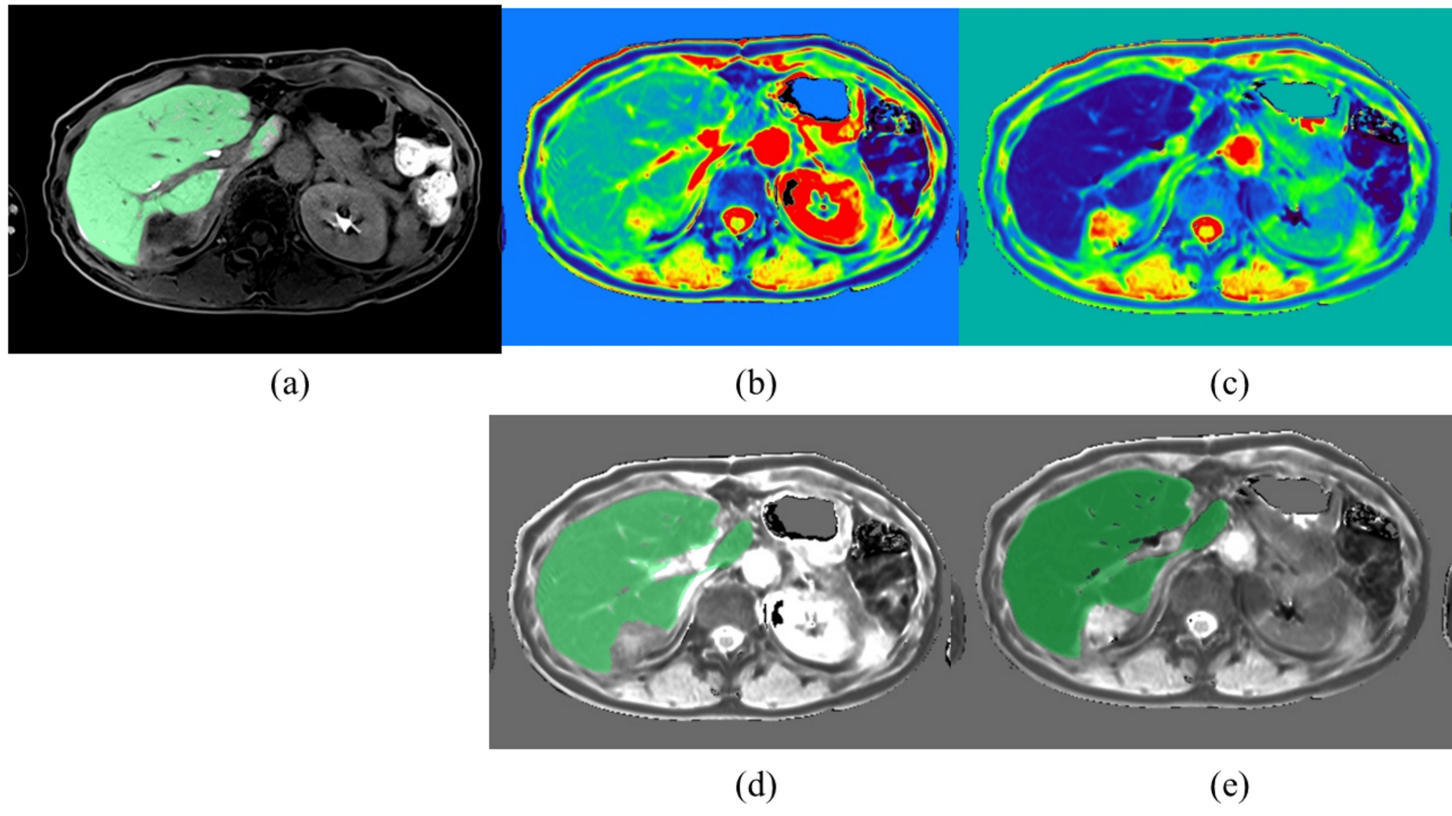
Axial cut MR images illustrate the whole liver T1 value measurement method. (a) ROI was set on the entire liver where the expected tumor and major vessels in the hepatobiliary phase (green area) were copied. (b) Pre-contrast T1 map (color map). (c) Post-contrast T1 map (color map). (d) ROI pasted on the pre-contrast T1 map (grayscale). (e) ROI pasted on the post-contrast T1 map (grayscale). MR: magnetic resonance; ROI: region of interest

The region of interest (ROI) was set on the whole liver where the expected tumor and major vessels in the hepatobiliary phase were copied, and the ROI was pasted on the T1 map. After the T1 value at each voxel was measured (Figure 2), the indices were calculated using the following formulas: ΔT1=Σ voxel (T1 pre-contrast−T1 post-contrast), T1 sum=Σ voxel (T1 pre-contrast or T1 post-contrast), T1 mean=Σ voxel (T1 sum/total numbers of voxel), and ΔT1 ratio=(T1 pre-sum−T1 post-sum)/T1 pre-sum.

The whole liver's volume was determined to evaluate the relationship between the liver volume and FLR. The volume was calculated semi-automatically based on the hepatobiliary phase by setting a threshold value and then excluding the major vessels and tumors. The following liver functional parameters of the GSA scintigraphy were calculated: HH15, representing the retention of the tracer in the blood; LHL15, representing the uptake of the tracer in the liver; and LU15, representing the cumulative liver uptake of the tracer between 15 and 16 min after injection. The parameters were calculated using the following formulas: HH15=heart count at 15 min/heart count at 3 min, LHL15=liver count at 15 min/sum of the liver and heart counts at 15 min, LU15=(total injection dose)×100%, where C(t) is the time-activity curve for the liver. The total injected dose was calculated as the difference in the radioactivity of the syringe before and after injection [17].

Results of the conventional FLR indices, including the ICGR15, CPC, MELD, ALBI, and ALICE, were obtained and calculated from the patient's medical records [4,18].

Statistical analysis

Data are presented as the mean±standard deviation. The Pearson product-moment correlation coefficient was used to evaluate the correlation between the GSA scintigraphy indices (HH15, LHL15, and LU15) and the EOB-MRI indices (T1 mean, T1 pre-sum, T1 post-sum, ΔT1, and ΔT1 ratio). In addition, the GSA scintigraphy and EOB-MRI indices were used to evaluate the correlation between the liver volume and conventional FLR indices by the Pearson product-moment correlation coefficient (r). The correlation strengths were determined r as follows: |r|≥0.7, strong; 0.7>|r|≥0.4, moderate; 0.4>|r|≥0.2, weak; and 0.2>|r|, no correlation.

Examinations that estimate remnant liver function, such as the LU15, T1 pre-sum, and T1 post-sum, were evaluated to determine whether a significant difference was present between the classified categories using a student t-test or a Kruskal-Wallis test when there was a significant correlation. Conventional FLR indices were classified into the following categories based on previous reports: (1) ICGR15 <20%, ≥20%; (2) CPC A, B; (3) MELD <10, ≥10; (4) ALBI 1, 2a, 2b; and (5) ALICE 1, 2, 3 [2,4,19,20]. Receiver operating characteristic (ROC) curves were created for the classifications found to be significant from these results; subsequently, the sensitivity and specificity were calculated by the Youden index.

The relationship between the FLR indices and postoperative complication score was evaluated using Spearman's rank correlation coefficient. The postoperative complication score was classified by the Clavien-Dindo grading system [21]. If a significant correlation was observed, the difference between the two grades provided by the Clavien-Dindo grading system, grade 1 and ≥grade 2, was compared by the Mann-Whitney U test. All statistical analyses were performed using IBM SPSS Statistics for Windows, Version 28.0 (Released 2021; IBM Corp., Armonk, New York, United States).

## Results

Forty-three patients were enrolled in this study; the patients' diseases included hepatocellular carcinoma (12), metastatic tumor (23), cholangiocellular carcinoma (four), gallbladder carcinoma (two), and bile duct carcinoma (two). Of the 43 patients, 31 had no specific liver disease, and the underlying liver diseases of the remaining patients were hepatitis B (seven), hepatitis C (four), and alcoholic liver disease (one) (Table 1).

**Table 1:** Characteristics of the study sample. ICGR15: indocyanine green retention rate at 15 min; CPC: Child-Pugh classification; MELD: model for end-stage liver disease; ALBI: albumin-bilirubin; ALICE: albumin-indocyanine green evaluation; HCC: hepatocellular carcinoma; Mets: metastatic tumor; CCC: cholangiocellular carcinoma; GB Ca: gallbladder carcinoma; Bile duct Ca: bile duct carcinoma

Parameters		
Age (mean)		64
Sex	Male	24
	Female	19
ICGR15 (%)		11
CPC	A	41
	B	2
MELD score	<10%	42
	≥10%	1
ALBI score	1	25
	2a	9
	2b	9
ALICE score	1	19
	2	22
	3	2
Underlying liver disease	Viral hepatitis B	7
	Viral hepatitis C	4
	Alcoholic	1
	None	31
Disease	HCC	12
	Mets	23
	CCC	4
	GB Ca/Bile duct Ca	2/2
Surgery	Yes	30
	No	13
Clavien-Dindo grade	1	20
	2	4
	3a	6

Classification of conventional FLR

The mean ICGR15 for all the patients was 11.0±6.34%. Thirty-nine patients had <20%, and four patients had ≥20%. The numbers of patients classified into CPC A and B were 41 and 2, respectively. The mean MELD score of all the patients was 1.23±3.48, with 42 patients having a score of <10 and one patient having a score of ≥10. The mean ALBI grade of all the patients was −2.60±0.48, with 25, nine, and nine patients classified into grades 1, 2a, and 2b, respectively. The mean ALICE grade of all the patients was −2.15±0.40, with 19, 22, and two patients classified into grades 1, 2, and 3, respectively (Table 1).

Correlation between the T1 map and GSA scintigraphy

A significant weak correlation was observed between the T1 pre- and post-sum and the LHL15 (r=−0.36 and −0.38, p=0.02 and 0.01, respectively). The other combination of T1 mapping and GSA scintigraphy indices did not exhibit a significant correlation (Table 2).

**Table 2:** Correlation between T1 map and GSA scintigraphy indices. Correlation coefficient (p-value), *p<0.05 HH15: heart count ratio after 15 min compared to after 3 min; LHL15: ratio of liver count to the sum of heart and liver count after 15 min; LU15: cumulative liver uptake of the tracer from 15 to 16 minutes after injection of 99mTc-GSA; GSA: galactosyl serum albumin; 99mTc-GSA: technetium-99m-galactosyl serum albumin

	HH15	LHL15	LU15
T1 mean	−0.15 (0.35)	−0.21 (0.18)	−0.05 (0.77)
T1 pre-sum	0.24 (0.12)	−0.36* (0.02)	−0.29 (0.06)
T1 post-sum	0.16 (0.31)	−0.38* (0.01)	−0.25 (0.11)
ΔT1	0.24 (0.13)	−0.23 (0.15)	−0.23 (0.14)
ΔT1 ratio	0.05 (0.78)	0.19 (0.23)	0.08 (0.63)

Influence of the liver volume

A strong correlation was observed between the liver volume and T1 pre- and post-sum (r=0.86 and 0.76, p<0.001 and <0.001, respectively). A moderate correlation was observed between the liver volume and ∆T1 (r=0.66, p<0.001), and a significant weak correlation was observed with HH15 and LU15 (r=0.33 and −0.30, p=0.03 and 0.047, respectively) (Table 3).

**Table 3:** Correlations between the radiological functional liver reserve and liver volume. *p<0.05, **p<0.01 HH15: heart count ratio after 15 min compared to after 3 min; LHL15: ratio of liver count to the sum of heart and liver count after 15 min; LU15: cumulative liver uptake of the tracer from 15 to 16 minutes after injection of 99mTc-GSA; 99mTc-GSA: technetium-99m-galactosyl serum albumin

Radiological index	Correlation coefficient	P-value
HH15	0.33*	0.031
LHL15	−0.29	0.058
LU15	−0.31*	0.047
T1 mean	0.07	0.669
T1 pre-sum	0.86**	<0.001
T1 post-sum	0.76**	<0.001
ΔT1	0.67**	<0.001
ΔT1 ratio	−0.19	0.226

Correlation between the radiological and conventional FLR indices

The T1 mean showed a moderate correlation with the ALBI and ALICE grades (r=0.58 and 0.49, p<0.001 and <0.001, respectively) and a weak correlation with the CPC (r=0.36, p=0.02). The T1 post-sum showed a moderate correlation with the ALBI grade (r=0.46, p=0.002) and a weak correlation with the ALICE grade (r=0.38, p=0.01). A mild correlation was observed between the ∆ T1 ratio and the CPC and ALBI and ALICE grades (r=−0.4, 0.58, and −0.55, p=0.008, <0.001, and <0.001, respectively). A significant weak correlation was observed between the HH15 value and ALICE grade (r=−0.33, p=0.03) and between the LU15 and ICGR15 values (r=−0.32, p=0.04) and the MELD score (r=−0.34, p=0.03) (Table 4).

**Table 4:** Correlations between radiological and conventional functional liver reserve indices. Correlation coefficient, (p-value), *p<0.05 ALBI: albumin-bilirubin; ALICE: albumin-indocyanine green evaluation; CPC: Child-Pugh classification; HH15: heart count ratio after 15 min compared to after 3 min; ICGR15: indocyanine green retention rate at 15 min; LHL15: ratio of liver count to the sum of heart and liver count after 15 min; LU15: cumulative liver uptake of the tracer from 15 to 16 minutes after injection of 99mTc-GSA; 99mTc-GSA: technetium-99m-galactosyl serum albumin; MELD: model for end-stage liver disease

	ICGR15	CPC	MELD score	ALBI score	ALICE score
HH15	−0.11 (0.50)	−0.18 (0.24)	−0.04 (0.80)	−0.26 (0.09)	−0.33* (0.03)
LHL15	−0.28 (0.07)	−0.10 (0.52)	−0.28 (0.07)	−0.11 (0.49)	−0.10 (0.51)
LU15	−0.32* (0.04)	0.14 (0.36)	−0.34* (0.03)	0.12 (0.46)	−0.06 (0.73)
T1 mean	0.08 (0.60)	0.36* (0.02)	0.07 (0.67)	0.58* (<0.001)	0.49* (<0.001)
T1 pre-sum	−0.05 (0.75)	−0.10 (0.54)	0.23 (0.15)	0.17 (0.28)	0.09 (0.57)
T1 post-sum	0.12 (0.44)	0.15 (0.33)	0.20 (0.21)	0.46* (0.002)	0.38* (0.01)
ΔT1	−0.20 (0.21)	−0.30 (0.05)	0.18 (0.26)	−0.16 (0.32)	−0.21 (0.17)
ΔT1 ratio	−0.26 (0.09)	−0.40* (0.008)	−0.06 (0.72)	0.58* (<0.001)	−0.55* (<0.001)

Evaluating the methods for estimating remnant liver function, such as the LU15 and T1 sum, demonstrated a significant correlation between the LU15 and ICGR15 values and the T1 post-sum and ALBI and ALICE grades. The LU15 values <20% and ≥20% were 24.75 and 19.88, respectively, which was significant (p<0.001). The T1 post-sum values of the ALBI grades 1, 2a, and 2b were 2.07, 2.5, and 2.8×107, respectively; a significant difference was observed between grades 1 and 2b (p=0.006). The T1 post-sum values of the ALICE grades 1, 2, and 3 were 2.06, 2.46, and 3.04×108, respectively; a significant difference was observed between grades 1 and 2 (p=0.02).

**Figure 3:**
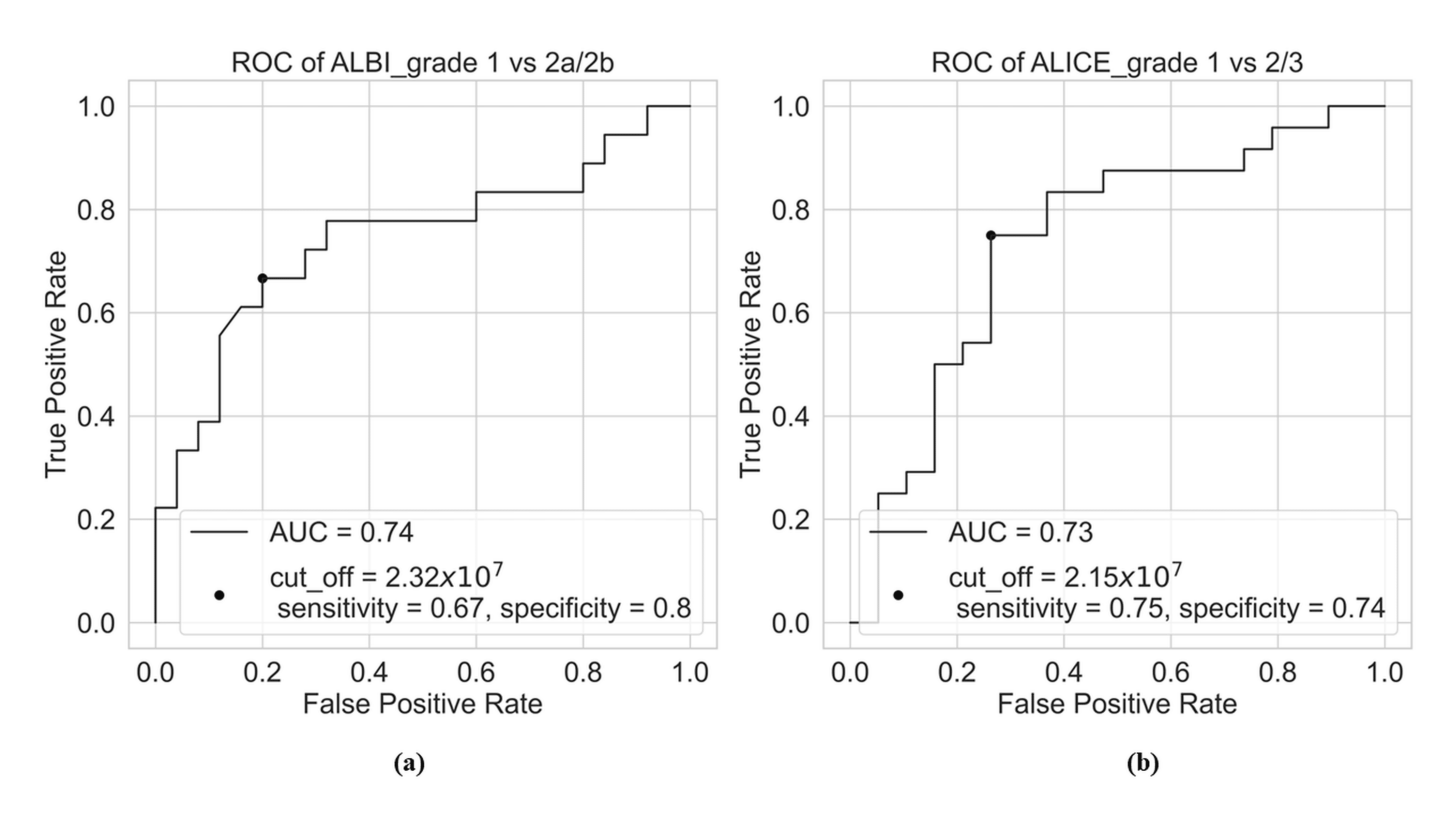
ROC curve analysis for classifying ALBI and ALICE indices into grades 1 and 2 or above. (a) Az value distinguishing ALBI grades 1 and 2a or 2b was 0.74 if the cutoff value was 2.32×107, sensitivity was 0.67, and specificity was 0.8. (b) Az value distinguishing ALICE grades 1 and 2 or 3 was 0.73 if the cutoff value was 2.15×107, sensitivity was 0.75, and specificity was 0.74. ROC: receiver operating characteristic; ALBI: albumin-bilirubin; ALICE: albumin-indocyanine green evaluation

The Az value distinguishing ALBI grades 1 and 2a or 2b was 0.74 (95% CI: 0.58-0.90) if the cutoff value was 2.32×107; the sensitivity was 0.67 and the specificity was 0.8 (Figure 3a). In contrast, the Az value distinguishing between ALICE grades 1 and 2 or grade 3 was 0.74 (95% CI: 0.58-0.89) if the cutoff value was 2.15×107; the sensitivity was 0.75 and the specificity was 0.74 (Figure 3b).

Postoperative complications, classified using the Clavien-Dindo grading system, occurred in 20, four, and six cases in grades 1, 2, and 3, respectively. No indices showed significant differences between grade 1 and Grades 2 or 3.

## Discussion

This study's findings demonstrated that the T1 post-sum can evaluate FLR; therefore, it can be used to estimate remnant liver function. Previously, authors have reported that only LU15 could evaluate remnant liver function while considering focal liver function and simulating liver resection [10]. The T1 post-sum showed a stronger correlation between the LU15 values and the conventional FLR indices in cases of relatively maintained liver function, such as in patients with CPC A.

The T1 sum strongly correlates with liver volume regardless of whether the measurement is performed before or after contrast enhancement. This correlation is more robust compared with that of GSA scintigraphy, possibly because of spatial resolution differences in each modality and the uptake mechanisms, i.e., EOB is taken up by the organic anion transporting polypeptide (OATP) 1B3 [22], while GSA binds to the asialoglycoprotein receptors on the membranes of hepatocytes [17]. Furthermore, GSA underestimates the function of the liver's left lobe and overestimates that of the right lobe [23]. Considering that a significant factor in surgical decisions is remnant liver volume, EOB-MRI is more suitable for evaluating remnant liver function compared with GSA scintigraphy [10,11,24].

This study's findings clarified that surrogate values, such as the T1 mean and ΔT1 ratio, correlate more strongly with conventional functional liver indices compared with the T1 sum. Representative values, such as average value and T1 shortening ratio, have been previously reported to correlate well [11-16]. No previously reported studies have used the sum of the T1 values for each pixel for the total amount of the entire liver. A surrogate value is based on the premise that the function of the entire liver is homogenous. Therefore, if the ROI is set on a locally strong fibrosis area in inhomogeneous parenchyma, the liver function may be underestimated [8].

The EOB-MRI and ICGR15 values showed weak correlations with the FLR indices, partially because they have the same transporter, OATP1 [22,25]. Kim et al. have reported that EOB-MRI's signal intensity is a better predictor of postoperative complications compared with ICGR15 results [6]. However, this study's findings showed no significant difference in the FLR indices between patients with and without complications; these results contradict those reported by Kim et al. [6]; this is perhaps because the liver function of the patients in this study was better compared with their patients.

Furthermore, the T1 post-sum significantly correlated with both the ALBI and ALICE grades. ALBI and ALICE grades can further stratify CPC A, which is indicated for surgery [4,18]. Therefore, the T1 post-sum may allow for the subclassification of patients' eligibility for surgery. The ALBI and ALICE grades are indicators based on albumin and are likely strongly influenced by the serum albumin levels. Albumin and bilirubin have been reported as confounding factors for the contrast effect in the hepatobiliary phase; therefore, the T1 post-sum can be used as an index of FLR [26].

This study's limitations include that it used retrospective data and the number of participants included was small. However, this study's results are valuable in that they show the efficacy of the T1 post-sum index obtained using EOB-MRI by comparing it with the conventionally used FLR indices. Unlike several previous studies, this study successfully evaluated the estimation of remnant liver function. In the future, a reliable cutoff T1 post-sum value should be set by collecting data from more cases. Another limitation is that the T1 post-sum may be affected by the contrast media in the bile duct, causing a weaker correlation between the liver volume and the T1 pre-sum. However, we suggest that this limitation can be ignored because the T1 post-sum correlates strongly with the liver volume.

## Conclusions

Representative indices, such as the T1 mean and ∆T1 ratio, show better relationships with conventional FLR indices compared with volumetric radiological indices. The T1 post-sum, which is the sum of the T1 values for each pixel of the whole liver in the EOB-MRI's hepatobiliary phase, can be used as an index of the FLR.
